# Primary Central Nervous System Lymphoma Mimicking the Presentation of Pituitary Apoplexy: A Case Report

**DOI:** 10.7759/cureus.50912

**Published:** 2023-12-21

**Authors:** Joao Meira Goncalves, Patricia Polónia, Josué Pereira, Pedro Alberto Silva

**Affiliations:** 1 Neurosurgery Department, Centro Hospitalar Universitário de São João, Porto, PRT; 2 Faculty of Medicine, University of Porto, Porto, PRT; 3 Neurosciences Centre, Hospital CUF, Porto, PRT

**Keywords:** non-hodgkin’s lymphoma, sellar, primary cns lymphoma, pituitary apoplexy, primary central nervous system lymphoma (pcnsl)

## Abstract

Pituitary lymphoma is one of the rare variants of primary central nervous system lymphoma (PCNSL), mostly arising due to the metastatic spread of systemic lymphoma.

We report the case of a 69-year-old woman who initially presented to her family physician with a headache but without any other symptoms. The MRI scan revealed a sellar mass consistent with a pituitary macroadenoma. When the patient was referred to our hospital, two weeks later, the symptoms had progressed, comprising complete right-sided ophthalmoplegia and ptosis, with left-sided amaurosis.

A repeat MRI revealed an increased size of the sellar mass, consistent with pituitary apoplexy. A right pterional craniotomy with partial resection of the mass was performed and an intraoperative frozen section biopsy was carried out. The final pathology revealed diffuse large B-cell lymphoma. A systemic follow-up including a whole-body CT, bone marrow aspiration, and cerebrospinal fluid studies ruled out any systemic manifestation, and the patient was HIV-negative. The patient underwent treatment with methotrexate, cytarabine, thiotepa, and rituximab for PCNSL.

Although rare, PCNSL can mimic pituitary apoplexy, which needs to be considered if conservative therapy or surgery is to be offered to a patient with a radiological and clinical diagnosis of pituitary apoplexy.

## Introduction

Primary central nervous system lymphomas (PCNSLs) are highly aggressive tumors. They are extranodal non-Hodgkin’s lymphomas (NHLs) and by definition manifest themselves in the brain, eyes, spinal cord, or leptomeninges, without systemic involvement at least at the time of primary diagnosis. This type of tumor can develop in both immunosuppressed or immunocompetent individuals but is thought to be rare in the second group, representing 4% to 6% of all extranodal lymphomas and accounting for 4% of all intracranial neoplasms [1]. The vast majority of PCNSL consists of diffuse large B-cell lymphomas (90%), and if we take into account their biological characteristics, they are extremely similar to systemic diffuse large B-cell lymphomas. Treatment for PCNSL has advanced gradually with relatively improved survival rates (almost 40%), but relapse is common, and long-term survival remains poor [2-4].

## Case presentation

In September 2020, a 69-year-old immunocompetent woman, with a previous history of parietooccipital parasagittal meningioma (WHO grade I) treated with surgery (and no recurrence) presented to her family physician reporting a right frontotemporoparietal headache without alarm signs, which had been developing over days. The neurological examination yielded no deficits. Her brain CT revealed an enhancing mass centered in the sellar/suprasellar region, leading to a suspicion of macroadenoma measuring 1.97 × 1.97 × 2.06 cm (anterior-posterior × transverse × cranial-caudal), and she was referenced to the pituitary oncology group at our tertiary university hospital. Endocrine function was normal and conservative treatment was advocated at that time.

Two weeks later, the patient presented at the emergency department with slowly progressive symptoms of binocular blurry vision, with an associated partial drooping of the right eyelid.

On initial physical examination, she was afebrile with stable vital signs. There was no altered mental state. On ophthalmic assessment, right-sided partial ptosis was noted. There was a mild anisocoria, with the right pupil being larger than the left; both were reactive to light. Adduction and upward and downward gaze were limited on the right eye, with associated diplopia in levoversion, supraversion, and infraversion, consistent with a third cranial nerve palsy. She had no symptoms suggestive of intracranial hypertension. The remainder of the neurological examination was normal with the absence of any alterations in visual acuity.

Blood work revealed multiple changes in her pituitary endocrine status, as shown in Table 1.

**Table 1:** Endocrine function. Analyses were conducted the morning after the event. This table depicts the dysregulation of hormone values, including elevated prolactin concentrations, decreased TSH levels, reduced free T4 levels, elevated morning cortisol concentrations, and elevated IGF-1 levels. TSH = thyroid-stimulating hormone; IGF-1 = insulin-like growth factor 1; ACTH = adrenocorticotropic hormone

Tests	Results	Normal range
Prolactin	30.6 ng/mL	4.8–23.3 ng/mL
TSH	0.04 mIU/L	0.35–4.94 mIU/L
Free T4	0.63 ng/dL	0.7–1.48 ng/dL
Morning cortisol	20.7 g/dL	6.2–19.4 μg/dL
IGF-1	207 ng/mL	51–187 ng/mL
Morning ACTH	40.8 ng/L	<63.3 ng/L

The patient’s visual acuity was stable. She screened positive for COVID-19 and was admitted to a specific surveillance cohort according to contemporary hospital guidelines.

In the following days, she remained stable as corticosteroids and levothyroxine were administered (for symptom relief and stabilization of hormonal deficiencies). An MRI confirmed a relatively homogenous, contrast-enhancing, large sellar and suprasellar mass, with only minimal signal heterogeneities, and no clear pattern on diffusion-weighted imaging (DWI) to confirm pituitary apoplexy.

After a thorough discussion within the multidisciplinary team, it was decided to postpone surgery, given the stable clinical picture, the new COVID-19 infection diagnosis, and its relative incompatibility with a transsphenoidal approach. The patient was discharged, subject to quarantine and close outpatient follow-up.

**Figure 1:**
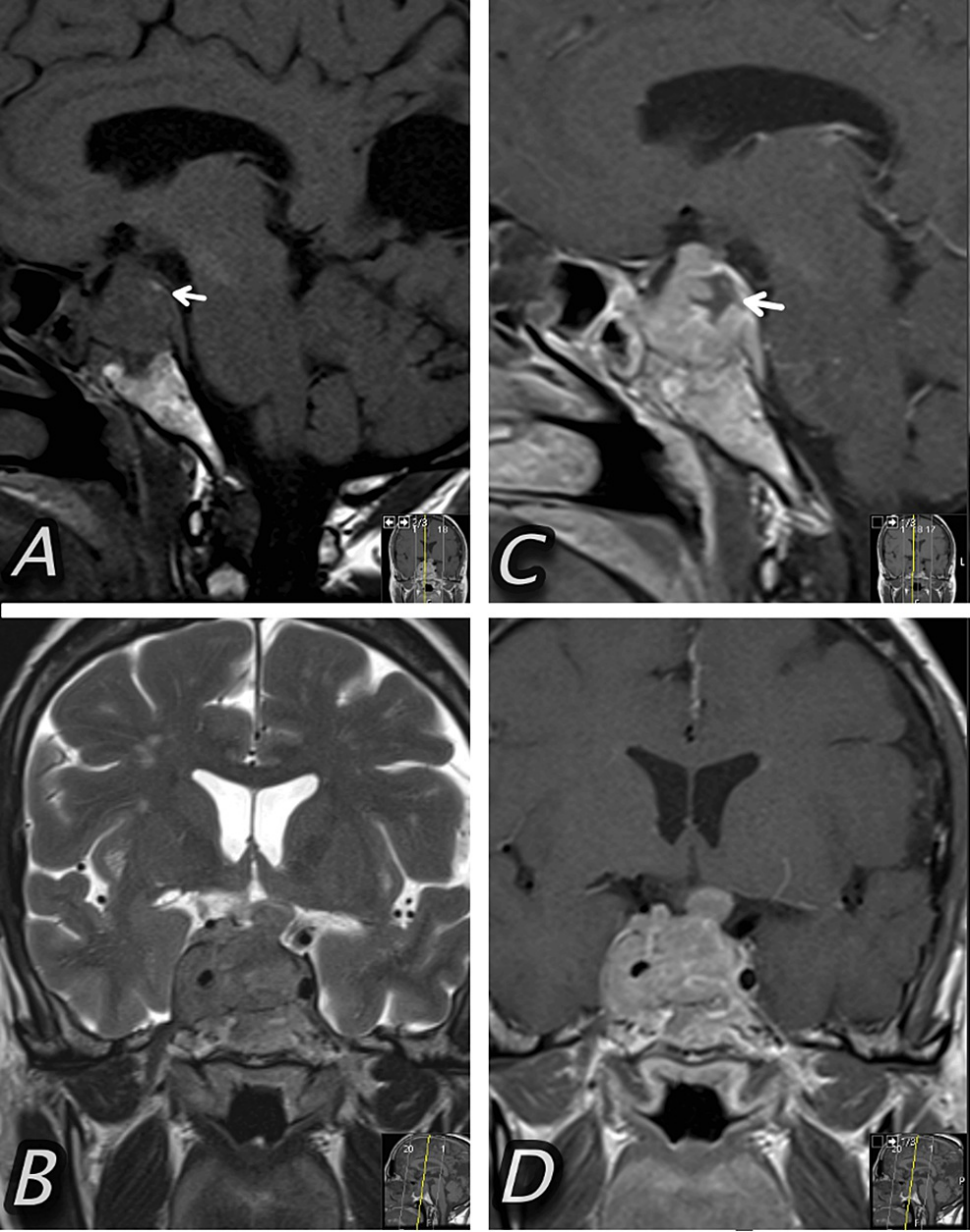
Brain MRI. (A) Sagittal T1-weighted image (WI) showing the large sellar lesion and the induced bone remodeling of the sphenoid; a small region of a high-intensity signal within the mass (small arrow) is seen that could correspond to blood products. (B) Coronal T2 WI showing a lobulation in the cranial aspect of the lesion that compresses the optic chiasm (not present in the previous MRI); it also shows an invasion of the right cavernous sinus. (C and D) Contrast-enhanced T1 WI sagittal and coronal, respectively. The mass enhances after contrast and it is possible to define areas of necrotic/cystic degeneration (small arrow).

Three weeks later, she was readmitted to the emergency department. She developed complete right-sided ophthalmoplegia and full right-sided ptosis. Her left eye had normal motility. Both pupils were mydriatic and non-reactive to dim light. She was able to count fingers with the right eye but she had no light perception on the left eye. A new brain MRI confirmed an increase in the size of the cellular mass, and it was interpreted as an “aftershock” pituitary apoplexy (Figure 1). Her COVID-19 status remained positive.

The patient was operated on through a right pterional craniotomy with partial resection of what was thought to be a pituitary adenoma, with spontaneous necrosis. An intraoperative frozen section biopsy was performed. Preliminary pathology of the cellular tissue was consistent with lymphoma.

**Figure 2:**
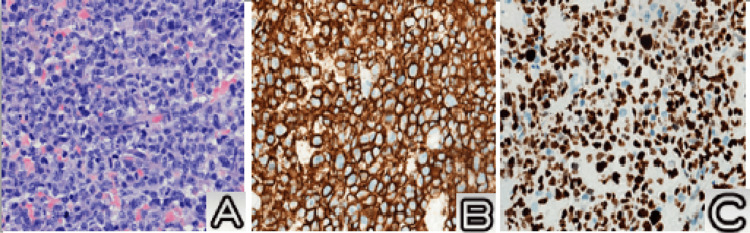
Diffuse large B-cell lymphoma. (A) Diffuse infiltration by atypical large lymphoid cells (hematoxylin and eosin, 400×). (B) Diffuse CD20 expression in neoplastic cells (400×). (C) High Ki-67 proliferation index (400×).

The final pathological examination demonstrated diffuse infiltration by atypical large lymphoid cells, with moderate cytoplasm and large irregular nuclei with open chromatin and prominent nucleoli. Numerous mitotic figures and apoptotic bodies were identified. On immunohistochemistry, neoplastic cells showed diffuse and strong expression of the B-cell marker CD20. Furthermore, diffuse immunoreactivity for MUM1, Bcl 2, and CD5 was observed in neoplastic cells. No expression of CD3 or Bcl 6 was observed. The Ki-67 proliferation index was higher than 90%. In-situ hybridization for Epstein-Barr virus-encoded RNAs was negative. A diagnosis of diffuse large B-cell lymphoma was made (Figure 2).

A contrast‐enhanced CT of the neck/chest/abdomen/pelvis and bone marrow biopsy excluded any systemic involvement. Full blood count parameters were normal and there was no detectable serum monoclonal protein to suggest a concurrent low‐grade lymphoma.

The patient was assigned to a chemo-immunotherapy regimen (MATRix regimen - four cycles, with each cycle administered every three weeks), consisting of methotrexate, cytarabine, thiotepa, and rituximab [3,4]. A reassessment CT scan of the head after the first week of therapy revealed a reduction in tumor size.

At follow-up one week later, on the CT scan, the patient had right-sided ptosis with right-sided amaurosis. She could see shapes and colors in her left eye, with no additional deficits. Unfortunately, she died three months later due to sepsis.

## Discussion

We report a rare presentation of PCNSL, as a large sellar and suprasellar mass with right cavernous sinus involvement, that began rather insidiously but later precipitated the abrupt onset of a complete third cranial nerve palsy of the right eye and left-sided amaurosis, mimicking pituitary apoplexy.

To our knowledge, there are only three known cases of PCNSL involving the pituitary gland presenting with acute neuro-ophthalmic findings [3,5-7]. Pituitary masses are being identified with increasing frequency due to the progressive improvement of endocrine tests and imaging techniques. Pituitary adenoma accounts for 10-15% of all intracranial neoplasms and represents the most common cause of mass in the sella [8].

PCNSL is characterized by its hypercellularity, so it is mainly iso- or hyperdense on unenhanced CT. The most sensitive imaging modality in the diagnosis of PCNSL is MRI with gadolinium contrast. Within the brain parenchyma, it most often presents with homogenous contrast enhancement characterized by well-defined boundaries. Mild surrounding edema is frequently present. A decreased signal on T2-weighted MRI and restricted diffusion on DWI are other features [9]. Necrotic/cystic lesions may be more common in HIV patients suffering from PCNSL but are uncommon in immunocompetent individuals [10,11].

Even though the literature describes that rapid improvement in clinical symptoms and radiographic features due to corticosteroids is common, this did not occur in our case, as the lesion had progressed since the first admission when the patient was started on corticosteroids.

Fluorodeoxyglucose (FDG) positron emission tomography (PET) may be helpful in distinguishing entities in the differential of a sellar mass, particularly when lymphoma is considered. Extremely high tumor FDG uptake relative to normal gray matter is suggestive of PCNSL. FDG-PET has an important role in PCNSL staging as well [10].

Non-functioning pituitary tumors typically emerge with visual compromise, including diminishing of the visual fields and reduced acuity. Signs of hypopituitarism due to pituitary stalk compression and non-specific symptoms, such as headache, are usually present. Pituitary lymphomas usually arise with symptoms of anterior pituitary hormone dysfunction [8].

Treatment of PCNSL includes radiation therapy and chemotherapy, often in combination with steroids and targeted therapy. Surgery is generally limited to stereotactic biopsy. No survival value of subtotal or gross total resection has been reported in retrospective studies. This may be attributed to the infiltrative and multifocal nature of PCNSL [1]. The importance of decompressive surgery compared to biopsy alone in PCNSL treatment is yet to be evaluated. In our case, conservative therapy with corticosteroids did not delay the progression of the disease. Cranial nerve palsy and visual loss prompted aggressive surgical resection. Although PCNSL often shrinks with the appropriate therapy, compression of the optic chiasm and optic nerves with acute visual deterioration may require emergent surgical intervention [9,12].

## Conclusions

We highlight that PCNSL can mimic pituitary apoplexy, which needs to be considered if conservative therapy or surgery is to be offered to a patient with the radiological and clinical diagnosis of pituitary apoplexy. When considering this type of entity, an intraoperative frozen section may be useful. Because sellar lymphoma often responds to medical treatment, complete resection is not always required and partial removal to decompress the optic chiasm may be enough.
